# An Updated Review of Randomized Clinical Trials Testing the Improvement of Cognitive Function of *Ginkgo biloba* Extract in Healthy People and Alzheimer’s Patients

**DOI:** 10.3389/fphar.2019.01688

**Published:** 2020-02-21

**Authors:** Haolong Liu, Min Ye, Hongzhu Guo

**Affiliations:** ^1^ School of Pharmaceutical Sciences, Peking University, Beijing, China; ^2^ Beijing Institute for Drug Control, NMPA Key Laboratory for Quality Evaluation of Traditional Chinese Medicine (Traditional Chinese Patent Medicine), Beijing Key Laboratory of Analysis and Evaluation on Chinese Medicine, Beijing, China

**Keywords:** *Ginkgo biloba* L., Alzheimer’s disease, cognitive function, clinical trials, EGb 761^®^

## Abstract

Alzheimer’s disease (AD) is a common neurodegenerative disease, mainly manifested by cognitive dysfunction. It seriously reduces the quality of life, and there is no ideal treatment strategy in clinical practice. Clinical studies on the treatment of AD with *Ginkgo biloba* L. leaf extract (EGb) have been reported since 1980s, and many clinical studies have been carried out during the following 30 years. However, the benefits of EGb on the treatment of AD are still controversial. In this review, we collected the clinical trial reports of EGb on cognitive function from Pubmed, Elsevier, Europe PMC, and other database since the 1980s. Through analysis and comparison, we consider that EGb may be able to improve the cognitive function in patients who suffered from mild dementia during long-term administration (more than 24 weeks) and appropriate dosage (240 mg per day). The main evidences and existing problems of the negative and positive experimental results were summarized.

## Introduction

Alzheimer’s disease (AD) is one of the age-related cognitive decline. It is a main challenge of mental health research (Tian et al., 2019), and is clinically characterized by cognitive dysfunction, neuronal injury, and synapse loss (Vanderstichele et al., 2019). Currently, there are approximately 50 million AD patients in the world (Bello-Medina et al., 2019). Although donepezil and memantine could delay AD symptom progression, side effects have been reported. Considering the number of AD patients and corresponding societal costs, plenty of clinical research has focused on the potential benefits of natural medicines.

*Ginkgo biloba* leaves are one of the popular herbal medicines, and they are recorded in the *Chinese Pharmacopoeia* (2015 edition). *G. biloba* leaves are well known in hypertension (Abdel-Zaher et al., 2018), ischemic myocardium (Zhang et al., 2016), and cerebral ischemia (Zhou et al., 2017). Remarkably, the extract of *G. biloba* leaves (EGb) attenuates the neuronal damage in animals (Li et al., 2017; Zhou et al., 2017) and *in vitro* (Yang et al., 2018). The chemical constituents of EGb are evaluated as follows: 22%–27% of flavone glycosides, 2.8%–3.4% of Ginkgolide A, B, and C, 2.6%–3.2% of bilobalide, and less than 5 ppm of ginkgolic acid (Chávez-Morales et al., 2017). Modern pharmacological studies have shown that the combination of extract of *G. biloba* and donepezil may be beneficial in the treatment of AD in rat and mice (Stein et al., 2015; Zhang et al., 2019). Furthermore, Ginkgolide B, one of the major active ingredients in EGb, can inhibit the neurotoxicity induced by β-amyloid (Shi et al., 2009; Li et al., 2013; Kaur et al., 2015; Gill et al., 2017).

However, whether EGb improves cognitive function in clinical study is a controversial question. Although a few available large-scale clinical trials suggest that EGb is relatively efficacious in delaying the progress of dementia, several other trials showed negative results. The present review searches large-scale clinical studies published in databases including Pubmed, ScienceDirect, and SpringerLink before 2019, and provides an analysis in the effective on cognitive function in EGb (**Tables 1A**, **B**).

**Table 1A:** The negative results of clinical trials of AD with EGb.

Study	Patients and gender female	Age (years) [mean(SD)]	Ginkgo biloba dose (mg/day)	Duration	Inclusion criteria	Outcome measures	Published date
Subhan and Hindmarch (1984)	8 F	32.0	120; 240; 600	One-dose	Healthy volunteers	CFF; CRT; LARS	1984
Schaffler and Reeh (1985)	8 M	27.3	80	2 w	Healthy volunteers	Complex choice test; battery of oculomotor and cardiorespiratory tests	1985
Lacomblez et al. (1990)	12 F	22.0	600	One-dose	Healthy volunteers	CFF; CRT; LARS; long memory (p <0.05)	1990
Stough et al. (2001)	26 F; 24 M	30.4	120	30 d	Healthy volunteers exclude head injury, intellectual developmental disability, neurological or psychiatric illness, current pregnancy, and current use of any other medication	Digit Symbol Substitution Test; Speed of Comprehension Test; Symbol Digit Modalities Test; Digit Span; Trail Making Test; Rey Auditory Verbal Learning Test; Inspection Time; working memory	2001
Kennedy et al. (2002)	15 F; 5 M	21.2	360	One-dose	Healthy undergraduate volunteers exclude heavy smoker and taking illicit social drugs	Cognitive measures; memory; attention; serial subtraction tasks; subjective mood measure	2002
Scholey and Kennedy (2002)	18 F; 2 M	19.9	120; 240; 360	One-dose	Healthy volunteers exclude taking other medication and heavy smoker	serial subtractions; speed of attention	2002
Dongen et al. (2003)	EGb: 68 F; 11 M Placebo: 36 F; 8 M	82.6 82.5	160; 240	24 w	a diagnosis of uncomplicated dementia age ≥ 50 years a score ranging from 8 to 23 on the SKT score absence of depression IQ > 80 absence serious comorbidity	Syndrom Kurz Test; Clinical Global Impression-2; Nürnberger Alters Alltagsaktivitäten Skala	2003
Mattes and Pawlik (2004)	EGb: 11 F; 10 M Placebo: 9 F; 9 M	24.1 23.1	184.5	13 w	Healthy volunteers 18–40 years of age body weight of 45.5–91 kg no known medical conditions not taking prescription medications no chemosensory abnormalities	Affect grid; POMS	2004
Elsabagh et al. (2005)	26 F; 26 M 19 F; 21 M	21.3 21.2	120	One-dose 6 w	Healthy volunteers exclude current use of psychoactive or anticoagulant medication exclude alcohol or drug dependence exclude pregnancy or lactation or current use of ginkgo	SoC; SWM; word presentation; picture presentation; mood rating scale; alcohol and caffeine diaries; PRM; SRM; word recall; picture recall; PASAT	2005
Schneider et al. (2005)	120 mg EGb: 84 F; 85 M 240 mg EGb: 96 F; 74 M Placebo: 90 F; 84 M	78.6 78.1 77.5	120; 240	26 w	a diagnosis of dementia of the Alzheimer’s type age ≥ 60 years duration of dementia symptoms at least 6 months Modified Hachinski Ischemic Score less than 4 Mini-Mental State Examination (MMSE) score of 10 to 24	ADAS-cog	2005
Burns et al. (2010)	EGb: 22 F; 24 M Placebo: 21 F; 26 M EGb: 54 M Placebo: 50 M	61.2 62.2 29.7 31.1	120	12 w	Healthy volunteers exclude taking cardiovascular, anticoagulant, antidepressant and antianxiety medication exclude any injury impaired cognitive tests	Woodcock-Johnson Psycho-Educational Battery-Revised	2006

AD, Alzheimer’s disease; EGb, Ginkgo biloba L. leaf extract; CFF, Critical Flicker Fusion test; CRT, Choice Reaction Time; LARS, Leeds Analogue Rating Scales; POMS, Profile of Mood States; SoC, Stockings of Cambridge; SWM, spatial working memory; PRM, pattern recognition memory; SRM, spatial recognition memory; PASAT, Paced Auditory Serial Addition Task; ADAS-cog, Alzheimer’s Disease Assessment Scale.

**Table 1B:** The positive results of clinical trials of AD with EGb.

Study	Patients and gender female	Age (years) [mean(SD)]	Ginkgo biloba dose (mg/day)	Duration	Inclusion criteria	Outcome measures included in research	Published date
Wesnes et al. (1987)	EGb: 8 F; 19 M Placebo: 12 F; 15 M	70.7 71.3	120	8 w; 12 w	Elderly patients have mild signs of impairment in everyday function on the Crichton Geriatric Rating Scale	Benton Visual Retention Test; Digit Span Test; Psychometric Test Battery	1987
Rai et al. (1991)	EGb: 8 F; 4 M Placebo: 12 F; 3 M	73.4 78.3	120	12 w; 24 w	Age ≥ 50 years Have signs of mild to moderate memory impairment classified by NINCDS-ADRDA	Folstein Mini-Mental State Examination; Kendrick Battery (p<;0.05); digit recall task (p<0.05)	1991
Maurer et al. (1997)	EGb: 5 F; 4 M Placebo: 4 F; 5 M	68.5 60.6	240	12 w	Age 50–80 years Hachinski Ischemic Score ≤ 4 Mild to moderate Alzheimer-type senile dementia Normal CT finding or a diffuse, possibly asymmetric, atrophy	SKT (p<0.05); Trailmaking test; ADCS	1997
Le Bars et al. (1997)	EGb: 65 F; 55 M Placebo: 72 F; 44 M	68	120	52 w	Age ≥ 45 years A diagnosis of uncomplicated dementia Mini-Mental State Examination score of 9 to 26 Global Deterioration Scale score of 3 to 6	ADAS-cog (p<0.05); GERRI (p<0.01)	1997
Kanowski et al. (1997)	EGb: 52 F; 27 M Placebo: 53 F; 24 M	EGb: 70.2 (F); 69.7 (M) Placebo: 72.0 (F); 66.4 (M)	240	24 w	Age ≥ 55 years SKT score of 6 to 18 MMSE score of 13 to 25	CGI (p<0.05); SKT (p<0.05); NAB	1997
Rigney et al. (1999)	14 F; 22 M	43.6	120; 150; 240; 300	2 d	Asymptomatic volunteers Good physical and mental health Free from concomitant medication	Short-term memory test (p<0.05); reaction times (p<0.05)	1999
Kennedy et al. (2000)	18 F; 2 M	19.9	120; 240; 360	One-dose	Health undergraduate volunteers Free from medication	Speed of attention (p<0.05); accuracy of attention; quality of memory (p<0.05); speed of memory (p<0.05)	2000
Le Bars et al. (2000)	EGb: 65 F; 55 M Placebo: 72 F; 44 M	68	120	26 w	Age ≥ 45 years A diagnosis of uncomplicated AD or multi-infarct dementia MMSE score of 9 to 26 Global Deterioration Scale score of 3 to 6	ADAS-cog (p<0.05); GERRI (p<0.01)	2000
Kanowski et al. (2003)	EGb: 72 F; 34 M Placebo: 70 F; 29 M	72	240	24 w	Age ≥ 55 years Suffered from dementia of the Alzheimer’s type or multi-infarct dementia SKT score of 6 to 18 MMSE score of 13 to 25	SKT (p<0.01); CGI (p<0.01); ADAS-cog (p<0.01)	2003
Mazza et al. (2006)	EGb: 13 F; 12 M Placebo: 16 F; 10 M	66.2 69.8	160	24 w	Brief Cognitive Rating Scale score of 3 to 5 Hachinski Ischemic Score < 4 IQ > 80	SKT (p<0.01); MMSE; CGI (p<0.01)	2006
Napryeyenko et al. (2007)	EGb: 143 F; 55 M Placebo: 142 F; 55 M	65 63	240	22 w	Age ≥ 50 years SKT score of 9 to 23 MMSE score of 14 to 25 ADAS-cog score of 17 to 35	NPI (p<0.01); SKT (p<0.01); GBS-ADL (p<0.01); HAMD (p<0.01)	2007
Napryeyenko et al. (2009)	EGb: 70 F; 34 M Placebo: 78 F; 32 M	66 64	240	22 w	Age ≥ 50 years SKT score of 9 to 23 MMSE score of 14 to 25 ADAS-cog score of 17 to 35 NPI score of 5 to 12	NPI (p<0.01); SKT (p<0.01); GBS (p<0.01)	2009
Ihl et al. (2011)	EGb: 139 F; 63 M Placebo: 133 F; 69 M	65	240	24 w	Age ≥ 50 years Cognitive score <35 SKT score of 9 to 23 MMSE score of 14 to 25 ADAS-cog score of 17 to 35 NPI score ≥ 5	NPI (p<0.01); SKT (p<0.01); ADCS-CGIC (p<0.01)	2011
Herrschaft et al. (2012)	EGb: 139 F; 61 M Placebo: 140 F; 62 M	65.1 64.9	240	24 w	Age ≥ 50 years Cognitive score <35 SKT score of 9 to 23 MMSE score of 14 to 25 NPI score ≥ 6	NPI (p<0.01); SKT (p<0.01); ADCS-CGIC (p<0.01)	2012
Ihl et al. (2012)	EGb: 109 F; 54 M Placebo: 111 F; 59 M	64.9 64.2	240	24 w	Age ≥ 50 years Total error score < 35 SKT score of 9 to 23 MMSE score of 14 to 25 NPI score ≥ 5	NPI (p<0.05); SKT (p<0.05); ADCS-CGIC (p<0.01)	2012
Hoerr and Nacu (2016)	EGb: 139 F; 61 M Placebo: 140 F; 62 M	65.1 64.9	240	24 w	Scored ≤ 35 in the Test for the Early Detection of Dementia with Differentiation from Depression SKT score of 9 to 23 NPI score ≥ 6	NPI (p<0.01); SKT (p<0.01)	2016
Rapp et al. (2018)	EGb: 69 F; 24 M Donepezil: 69 F; 28 M	84.4 84.2	240	12 m	Age ≥ 80 years MMSE score of 10 to 23 DemTect score ≤ 9 Scheltens index ≥ 2 Fazekas score ≤ 2	MMSE	2019

SKT, Syndrom Kurz Test; GERRI, Geriatric Evaluation by Relative’s Rating Instrument; CGI, Clinical Global Impression; NAB, Nüberger Alters-Beobachtungsskala; GBS-ADL, Gottfries–Bråne–Steen activities-of-daily-living; NPI, Neuropsychiatric Inventory; HAMD, Hamilton Rating.

Scale for Depression; ADCS-CGIC, Alzheimer’s Disease Cooperative Study—Clinical Global Impression of Change.

NINCDS-ADRDS, National Institute of Neurological and Communicative Disorders and Stroke-Alzheimer's Disease and Related Disorders Association; CT, computerized tomography.

## Negative Clinical Results on Cognitive Function

### Single-Dose Studies

A clinical study was conducted on the effect of EGb on cognitive function by Subhan in 1984 (Subhan and Hindmarch, 1984). Eight healthy female subjects were administered EGb or placebo, and the psychometric scale was tested after 1 h of administration. Only Sternberg technique was significantly improved with 600 mg EGb to placebo, and there was no difference in Critical Flicker Fusion test (CFF), Choice Reaction Time (CRT), and Leeds Analogue Rating Scales (LARS) among those groups. In 1985, Schaffler et al. (Schaffler and Reeh, 1985) showed EGb only improved complex choice reaction time in a clinical study.

Based on Subhan’s research, Lacomblez et al. (1990) designed a clinical study in healthy young female volunteers in 1990. The volunteers received a single dose of EGb (600 mg) or placebo, and a battery of tests were used after dosing. The results indicated that no statistical differences were observed on CFF and CRT. However, the long-term memory was improved with the followed treatment.

In 2002, Kennedy and Scholey collaborated on two clinical trials (Kennedy et al., 2002; Scholey and Kennedy, 2002). In Kennedy’s research, 20 healthy young volunteers received ginkgo, ginseng, ginkgo combined with ginseng, and placebo, respectively. The Cognitive Drug Research (CDR) computerized assessment battery and cognitive outcome factors were used for evaluating cognitive function. For CDR battery, a statistical difference was only observed in immediate word recall, numeric working memory, delayed word recalls, and picture recognition reaction time at 6 h after administration. Furthermore, the quality of memory and serial 7s responses was improved at 6 h after administration. However, other test items had no statistical difference between EGb group and placebo. In Scholey’s research, the dosage of EGb was adjusted to 120, 240, and 360 mg. In Serial Threes Task, a statistical difference was only observed in EGb group at 4 h after administration. There was no effect on Serial Seven Task at any time point after oral administration.

### Long-Term Studies

In 2001, 26 healthy young females and 24 healthy young males were enrolled in a 30-day clinical trial (Stough et al., 2001). Then the subjects were assessed on the neuropsychological tests. The tests included the Digit Symbol Substitution Test (DSST), Speed of Comprehension Test (SCT), Symbol Digit Modalities Test (SDMT), Digit Span; Trail Making Test (TMT), Rey Auditory Verbal Learning Test (AVLT), and Inspection Time (IT). The validated battery of tests was employed to evaluate attention, memory, and work performance. The results indicate that Digit Span Backwards, Working Memory Speed, and AVLT were improved in participants after administration, but the effect was not observed on any of the other tests.

In 2003, 214 participants were allocated to one of three treatments (160 mg/day of EGb, 240 mg/day of EGb, and placebo) (Dongen et al., 2003). All the participants received EGb for 12 weeks, then randomly allocated for their *Ginkgo* treatment or placebo for another 12 weeks. The score for Syndrom Kurz Test (SKT) and Nürnberger Alters Alltagsaktivitäten (NAA; Nuremberg Gerontopsychological Rating Scale for Activities of Daily Living) were not statistically significant after 24 weeks.

In 2004, 20 females and 19 males were supplemented for 13 weeks with 240 mg of EGb, then the alertness and chemosensory function was used to evaluate cognitive function (Mattes and Pawlik, 2004). The results showed that EGb could improve performance on the chemosensory, but was ineffective at taste and smell function. Quality control problems of the EGb tablets may result in ineffectiveness after administration.

In 2005, 52 university students were randomly divided into EGb and placebo group (Elsabagh et al., 2005). Another 40 students received EGb and placebo in long-term study. In both experiments, the performances were evaluated through sustained attention, episodic memory, executive function, and completed mood rating scales. Sustained attention and pattern recognition memory were improved after acute ginkgo treatment, but there were no effects on other tests. There were also no effects on any cognitive tests after 6-week treatment.

To determine the clinical efficacy of EGb in AD, outpatients were supplemented for 26-week treatment with EGb or placebo (Schneider et al., 2005). Finally, 513 outpatients, including 270 females and 243 males, were randomized to receive 120 mg/day of EGb or 240 mg/day of EGb or placebo for 26 weeks. The changes of Alzheimer’s Disease Assessment Scale—cognitive subscale (ADAS-cog) and Alzheimer’s Disease Cooperative Study—Clinical Global Impression of Change (ADCS-CGIC) between baseline and week 26 were applied for evaluation strategy. There were no significant differences between groups, and the trial did not show efficacy of EGb.

In 2006, Burns et al. published the results of their clinical experiments, which was a 12-week study assessing effects of EGb on cognitive abilities in healthy young (mean age 30.4 years) or older (mean age 61.7 years) adults (Burns et al., 2010). The effect of EGb was evaluated by a series of tests, including cognitive abilities test, chronometric test, and subjective well-being. There were no statistically significant differences on any tests in young participants.

## Positive Clinical Results on Cognitive Function

### Single-Dose Studies

In 1999, Rigney et al. designed a clinic study to evaluate the effect of EGb (Rigney et al., 1999). Thirty-one health volunteers randomly received 120 mg of EGb or 240 mg of EGb or placebo. The battery of test consisted of Steinberg Short Term Memory (SSTM), Linear Analogue Rating Scale (LARS), Leeds Sleep Evaluation Questionnaire (LSEQ), Scanning Task (STM), CFF, CRT, SCT, DSST, and Actigraphy. After administration of EGb, there were statistically significant differences in reaction time, motor reaction time, and CFF, and EGb were more pronounced for memory than attention.

In 2000, a study was designed to investigate the effects of acute doses of EGb on cognitive performance (Kennedy et al., 2000). Eighteen female and two male healthy college students (age 19–24 years) received 120, 240, and 360 mg of EGb or placebo. A tailored version of the CDR computerized assessment battery was used for measuring cognitive function as a consequence of ingestion of EGb of placebo. Compared with the placebo, cognitive function was improved in a number of assessment substances. It was noteworthy that a number of tests were performed dose-dependently after acute administration of EGb.

### Long-Term Studies

In 1987, outpatients including 20 females and 34 males (mean age 71.3 years) received 120 mg of EGb or placebo for 12 weeks (Wesnes et al., 1987). The test items included cognitive test buttery and automated psychometric test battery. The batteries comprised the following tests: the Benton Visual Retention Test, Immediate Word Recall, Number Matching Task, Rapid Visual Information Processing Task, CRT, DSST, and Word Recognition. To avoid the limitation and the one-sidedness of experimental results, all tests were combined and measured using two different techniques. The results indicated that EGb was superior to placebo in cognitive tests after administration for 12 weeks. The same results were repeated in another clinical trial in 1991 (Rai et al., 1991).

In 1997, nine females and nine males, aged 50–80 years and suffering from mild to moderate dementia of the Alzheimer type, were randomly divided into two groups. These patients were treated with a daily dose of 240 mg EGb or placebo for 3 months (Maurer et al., 1997). The SKT scores, Trailmaking test (ZVT), and ADAS were used to investigate the effect of EGb on the improvement of cognitive function. Among them, there was a significant difference in SKT test between the EGb group and the placebo group, while there was no significant difference in the other two tests. The SKT was particularly sensitive to changes in mild and moderate dementia, but ADAS showed lower sensitivity than SKT. Furthermore, ADAS showed similar trend with SKT scores.

In 1997, Kanowski et al. also evaluated the efficacy of EGb in 156 outpatients who suffered from dementia of the Alzheimer type (Kanowski et al., 1996). Patients were evaluated by Clinical Global Impressions (CGI), SKT, and Nüberger Alters-Beobachtungsskala (NAB), respectively. There were significantly differences between EGb and placebo.

Le Bars et al. published two trials of EGb for dementia in 1997 and 2000 (Le Bars et al., 1997; Le Bars et al., 2000). In 1997, 309 patients were enrolled in the study, and the primary outcome measures included ADAS-cog, Geriatric Evaluation by Relative’s Rating Instrument (GERRI), and CGIC. For the ADAS-cog and GERRI, there was a significant change observed in EGb group at the end point, and no difference was demonstrated in the CGIC. The same results were repeated in 2000.

Hoerr et al. reported the benefits of EGb in dementia of AD type in 2003, 2012, and 2016, respectively (Kanowski and Hoerr, 2003; Herrschaft et al., 2012; Hoerr and Nacu, 2016). In 2003, 205 patients were included in a trial, and the primary outcome measures were SKT, CGI, and NAB. The effects were statistically significant in SKT and CGI. The EGb showed a mild improvement of NAB in treatment group and placebo group, although there was no statistical difference between the groups. In 2012, the Neuropsychiatric Inventory (NPI) was added into the test item in order to investigate the benefit effect of EGb on neuropsychiatric symptoms (NPSs). The dosage of administration and course of treatment referred to the clinical trial in 2003. The results indicated that EGb alleviated NPSs in patients with dementia. The same results were repeated in another clinical trial in 2016.

In 2006, Mazza et al. designed a study to compare the therapeutic effect of EGb and donepezil in patients with AD (Mazza et al., 2006). The 76 patients were randomly divided into EGb group, donepezil group, and placebo group, respectively. Mini-Mental State Examination (MMSE), SKT, and CGI were important parameters in demonstrating the clinical efficacy of EGb in cognitive performance. Finally, EGb group and donepezil group showed a statistically significant difference compared with placebo. The results confirmed the efficacy of EGb in cognitive performance, comparable with donepezil clinical efficacy.

In 2007 and 2009, two large-scale clinical trials were designed to study the effect of EGb in cognitive performance by Napryeyenko (Napryeyenko and Borzenko, 2007; Napryeyenko et al., 2009). In 2007, 391 outpatients suffered from AD or vascular dementia (VaD). The patients in EGb group received 240 mg/day of EGb for 22 weeks. The NPI, SKT, and activities-of-daily-living subscale of Gottfries–Bråne–Steen (GBS-ADL) were used to evaluated the clinical efficacy of EGb, and there were significant differences in the test battery. The same results were repeated in another clinical trial in 2009, and EGb was safe and effective in the treatment of mild dementia caused by AD or VaD.

In 2011 and 2012, Ihl published two clinical studies, which were used to confirm the therapeutic effects of EGb on cognitive dysfunction in elder patients suffered from AD (Ihl et al., 2011; Ihl et al., 2012). In both researches, more than 400 outpatients were randomly divided into two groups, which were allocated to receive EGb (240 mg/day) or placebo for 24 weeks. In the result, SKT and NPI scores were improved by EGb, all secondary outcome measures were also improved, including ADCS-CGIC, Verbal Fluency Test, and Alzheimer’s Disease Activities-of-Daily-Living International Scale (ADL-IS). Furthermore, adverse event rates were not increased in EGb group. Therefore, EGb was significantly superior to placebo in the treatment of dementia with NPSs.

Recently, Rapp compared treatment outcomes to provide the safety and efficacy of EGb (Rapp et al., 2018). The 189 patients were randomly treated with EGb (240 mg/day) or donepezil (5 or 10 mg/day) for 12 months. There were no significant differences in cognitive decline measured with the MMSE over 12 months, and the last observation carried forward (LOCF) showed a mild favorable effect for EGb 761 over donepezil.

## Discussion

EGb has been used therapeutically for many years for symptoms such as cognitive decline, memory deterioration, and decreased alertness. In this review, we summarized the clinical studies from 1984 to 2018, aiming to evaluate the effects of EGb on the intervention of cognitive dysfunction. By comparing the above negative and positive results in **Tables 1** and **2**, we found several key factors that influenced the results.

**Table 2:** Consensus statements of EGb from the Asian Clinical Expert Group on Neurocognitive Disorders.

No	Consensus statement	Concrete content
1	Efficacy of EGb 761^®^ in AD, VaD, and BPSD	Best practice for the pharmacological treatment as follows:
		AD: AChEI, memantine, EGb
		VaD: AChEI, memantine, EGb, antiplatelet therapy
		BPSD: ChEI, nonpharmacological treatment, antipsychotics (off-label), memantine, SSRIs, sedatives, and EGb
2	Management of MCI	EGb may be considered for use in patients with MCI
3	How to use EGb	EGb can be used as a single agent, and allow sufficient time to take effect
4	The dosage	EGb at daily dose of 240 mg
5	Lack of efficacy or intolerance of standard drugs may warrant use of EGb	EGb was recommended to treat AD, VaD, and mixed dementia, when the patients unable to tolerate the side effects of standard treatments
6	Adjunctive therapies	EGb was one of the key management options adjunctive to standard pharmacological therapy for AD, VaD, and BPSD
7	Management of comorbidities	EGb played an important role in the management of co-morbidities, such as hypertension, in patients with AD, VaD, and BPSD
8	Does not appear to prevent dementia	EGb was not recommended for prevention of dementia
9	Well tolerated	EGb had a good tolerability profile in the treatment of MCI, AD, VaD, and BPSD
10	No overall increased bleeding risk	EGb appeared to be no overall added risk of bleeding
11	No significant interaction with anticoagulants or antiplatelet agents	EGb had been demonstrated no significant interaction with anticoagulants and antiplatelet agents

AD, Alzheimer’s disease; VaD, vascular dementia; BPSD, behavioral and psychological symptoms of dementia; MCI, mild cognitive impairment; AChEI, acetylcholinesterase inhibitors; SSRIs, Selective Serotonin Reuptake Inhibitors.

### Course and Dosage of Administration

Among the 12 negative results, the duration of administration was mostly single or short-term (less than 12 weeks), and only two experiments were conducted for long-term administration (more than 22 weeks). On the other hand, in the positive results, 11 studies were administered for longer than 22 weeks. In Ihl’s and Napryeyenko’s research, the changes of SKT score and NPI score were obviously observed in 12-week and 24-week. The differences between the EGb group and the placebo group were gradually expressed with the progress of treatment. Therefore, long-term study was more suitable to evaluate the therapeutic effect of EGb on cognitive dysfunction caused by AD.

Among the 11 negative results, 120 mg or less EGb was administered to treat patients in seven studies. However, 10 positive studies allocated to the treatments with 240 mg or more of EGb. Mattes got negative results, but found that the tablets contained only 65% of the expected concentration in re-analysis. Through literature review and analysis, Mattes found that the treatment can achieve better therapeutic effect when the dosage of EGb was more than 240 mg/day. This opinion was also confirmed in Hashiguchi’s research.

### Inclusion Criteria

Through the analysis of negative and positive results, the inclusion criteria play a key role. In the negative studies, nine clinical studies involved healthy young subjects. However, 13 clinical studies involved elder outpatients in positive research. In the meta-analysis published by Lijuan Jiang (Jiang et al., 2013), EGb was more suitable for the treatment of elderly patients, especially aged 60 to 75 years. Furthermore, several studies have also set strict rules about the inclusion criteria, including the MMSE, SKT, and NPI score.

### Outcome Measures

Through meta-analysis, Hyde thought that EGb was more suitable for the treatment of patients suffered from AD combined with mental disorder (Hyde et al., 2017). Hashiguchi further pointed out that SKT score was more applicable to evaluate patients with mild-moderate AD (Hashiguchi et al., 2015). During recent years, SKT score was used to evaluate the therapeutic effect in positive results.

In 2018, nine countries, including China, Germany, and Singapore, had jointly published a clinical guideline on the treatment of dementia mild cognitive impairment with EGb (Kandiah et al., 2019). In the guideline, several compelling evidences supported the inclusion of EGb as part of the treatment armamentarium for AD or VaD (**Table 2**). EGb showed encouraging efficacy in terms of improvement in cognition impairment and mental condition in patients with AD or VaD.

Through analysis and comparison, we consider EGb may be able to improve the cognitive function in patients suffering from mild dementia during administration (more than 24 weeks) and appropriate dosage (240 mg per day). However, there are some limitations in the studies that should be noted. AD is a disease with a long course; the effects of the drug may change over time. According to a recent research, aducanumab was deemed to indicate little chance of treatment efficacy in March 2019 (Howard and Liu, 2019), but the conclusion was overturned with the full 18 months of treatment in October 2019 (http://investors.biogen.com/news-releases/news-release-details/biogen-and-eisai-discontinue-phase-3-engage-and-emerge-trials). In our opinion, most clinical trials of EGb were less than 24 weeks. We suggest that clinical trials with longer treatment are more appropriate to demonstrate the efficacy of EGb. Furthermore, some key biomarkers such as apolipoprotein E4 (*APOE4*) were identified as a single greatest genetic risk factor for sporadic AD in clinical settings (Lin et al., 2018; Belloy et al., 2019; Berg et al., 2019). However, *APOE4* was not involved in the study of EGb. Therefore, the relationship between EGb and APOE was still a mystery; it may be a research direction in the future.

## Author Contributions

HG conceived the study. HL acquired the data and drafted the manuscript. HG and MY revised the manuscript. All authors read and approved the final manuscript.

## Funding

This study was supported by the Beijing Natural Science Foundation (No. 7194283) and the Beijing Postdoctoral Research Foundation (No. 2018-ZZ-069).

## Conflict of Interest

The authors declare that the research was conducted in the absence of any commercial or financial relationships that could be construed as a potential conflict of interest.
